# Supplementary material for: PPDPF alleviates hepatic steatosis through inhibition of mTOR signaling
Source: Nat Commun. 2021 May 24;12:3059. doi: 10.1038/s41467-021-23285-8 (PMC8144412; doi:10.1038/s41467-021-23285-8)
Supplement: Supplementary file 1 — Supplementary Information [file 41467_2021_23285_MOESM1_ESM.pdf]

Supplementary Information

PPDPF alleviates hepatic steatosis through inhibition of mTOR signaling

Ning Ma[1,10], Yi-Kang Wang[1,10], Sheng Xu[1], Qian-Zhi Ni[1], Qian-Wen Zheng[1,2], Bing Zhu[1], Hui-Jun Cao[1], Hao Jiang[1], Feng-Kun Zhang[1], Yan-Mei Yuan[1], Er-Bin Zhang[1], Tian-Wei Chen[1], Ji Xia[1], Xu-Fen Ding[1], Zhen-Hua Chen[3], Xiu-Ping Zhang[4], Kang Wang[3], Shu-Qun Cheng[3], Lin Qiu[1], Zhi-Gang Li[5], Yong-Chun Yu[6], Xiao-Fan Wang[7], Bin Zhou[8], Jing-Jing Li[1*] and Dong Xie[1,2,9*]

[1]CAS Key Laboratory of Nutrition, Metabolism and Food Safety, Shanghai Institute of Nutrition and Health, University of Chinese Academy of Sciences, Chinese Academy of Sciences, 200031 Shanghai, China.

[2] School of Life Science and Technology, ShanghaiTech University, 393 Middle Huaxia Road, Shanghai 201210, China.

[3] Department of Hepatic Surgery VI, Eastern Hepatobiliary Surgery Hospital, Second Military Medical University, 200438 Shanghai, China.

[4]Department of Hepatobiliary and Pancreatic Surgical Oncology, The First Medical Center of Chinese People's Liberation Army (PLA) General Hospital, Beijing, China.[5]Department of Thoracic Surgery, Section of Esophageal Surgery, Shanghai Chest Hospital, Shanghai Jiao Tong University, 200030 Shanghai, China.

[6]Shanghai Institute of Thoracic Oncology, Shanghai Chest Hospital, Shanghai Jiao Tong University, Shanghai, 200030, China.

[7]Department of Pharmacology and Cancer Biology, Duke University Medical Center, Durham, NC, 27705, USA.

[8]The State Key Laboratory of Cell Biology, CAS Center for Excellence on Molecular Cell Science, Shanghai Institute of Biochemistry and Cell Biology, Chinese Academy of Sciences, University of Chinese Academy of Sciences, Shanghai 200031, China.

[9] NHC Key Laboratory of Food Safety Risk Assessment, China National Center for Food Safety Risk Assessment, Beijing 100022, China.

[10]These authors contributed equally：Ning Ma, Yi-Kang Wang.

[*]Correspondence and request for materials should be addressed to Jing-Jing Li (tide7@163.com) or Dong Xie (dxie@sibs.ac.cn).

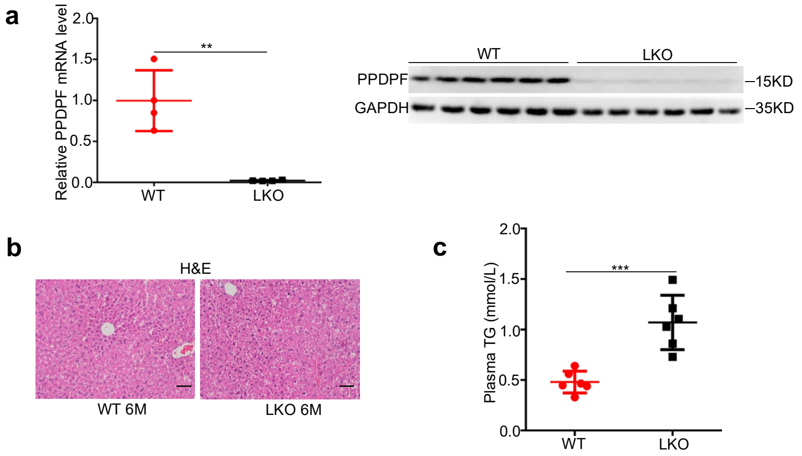

Supplementary Fig.1 The knockout efficiency of PPDPF and plasma TG in mice. **(a)** PPDPF expression level in the liver of WT (n=4) and PPDPF-LKO (n=4) mice at 8 months is measured by real-time qPCR and western blotting. The mRNA expression levels of the genes are normalized to that of 18s. Mean±SEM, ***p*=0.0019 by two tailed unpaired Student's *t*-test. **(b)** H&E staining of liver sections from WT and KO mice at 6 months. Scale bars,100um.**(c)** Plasma triglyceride (TG) contents from WT (n=6) and LKO (n=6) mice at 8 months. Mean±SEM, ****p*=0.0005 by two tailed unpaired Student's *t*-test. All experiments were repeated 3 times independently.

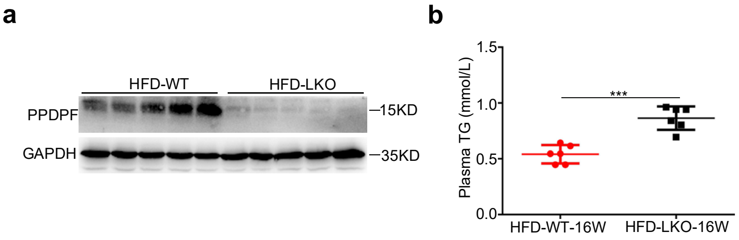

Supplementary Fig.2 The knockout efficiency of PPDPF and plasma TG in the mice fed HFD for 4months.(a) PPDPF expression level in the liver from WT and KO mice fed HFD for 4 months is measured by western blotting.**(b)** Results of plasma triglyceride (TG) tests of WT (n=6) and LKO (n=6) mice fed HFD for 4 months. Mean±SEM, ****p*=0.0001 by two tailed unpaired Student's *t*-test. All experiments were repeated 3 times independently.

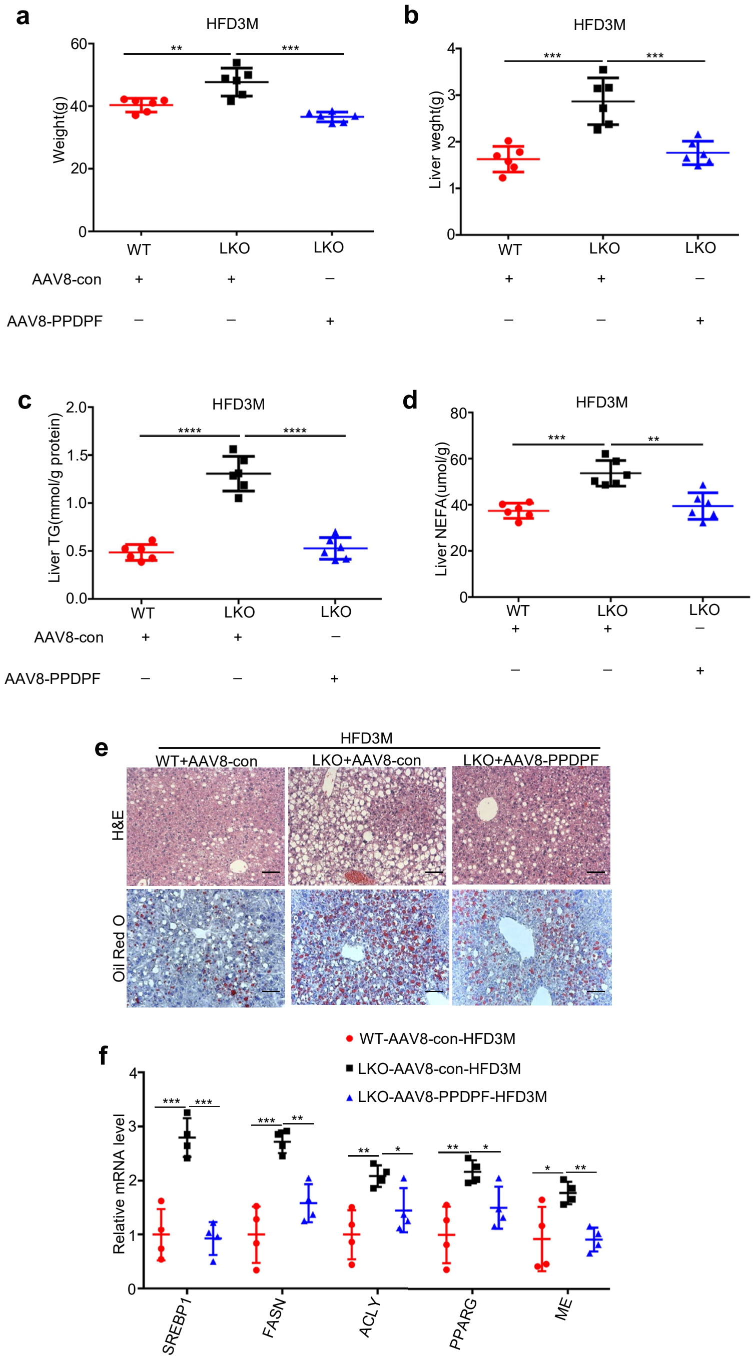

Supplementary Fig.3 AAV8-mediated reintroduction of PPDPF rescues PPDPF -null phenotype in PPDPF-LKO mice fed HFD for 3 months.(a-d) The body weight(a), liver weight(b), TG(c) and NEFA(d) for each group (WT-AAV8-con (n=6), LKO-AAV8-con (n=6) and LKO-AAV8-PPDPF (n=6)) after 3 months HFD feeding. Mean±SEM, (a) ***p*=0.0045 (LKO+AAV-con Vs WT+AAV-con), ****p*=0.0002 (LKO+AAV-PPDPF Vs LKO+AAV-con); (b) ****p*=0.0003 (LKO+AAV-con Vs

WT+AAV-con), ***p=0.0007 (LKO+AAV-PPDPF Vs LKO+AAV-con); (c) ****p<0.0001; (d) ***p=0.0001 (LKO+AAV-con Vs WT+AAV-con), **p=0.0015 (LKO+AAV-PPDPF Vs LKO+AAV-con) by two tailed unpaired Student's t-test. **(e)**Representative images of H&E and Oil Red O staining of liver sections from the mice injected with indicated adenovirus fed HFD for 3 months. Scale bars, 100 um.**(f)**The mRNA expression level of lipogenesis-related genes for each group (WT-AAV8-con (n=4), LKO-AAV8-con (n=4) and LKO-AAV8-PPDPF (n=4)) after 3 months HFD feeding. Mean±SEM, *SREBP1*: ***p=0.0009 ***p=0.0002, *FASN*: ***p=0.0009 **p=0.0014, *ACLY*: **p=0.0048 *p=0.0321, *PPARG*: **p=0.0061 *p=0.0242, *ME*: *p=0.0353 **p=0.0012 by two tailed unpaired Student's t-test. All experiments were repeated 3 times independently.

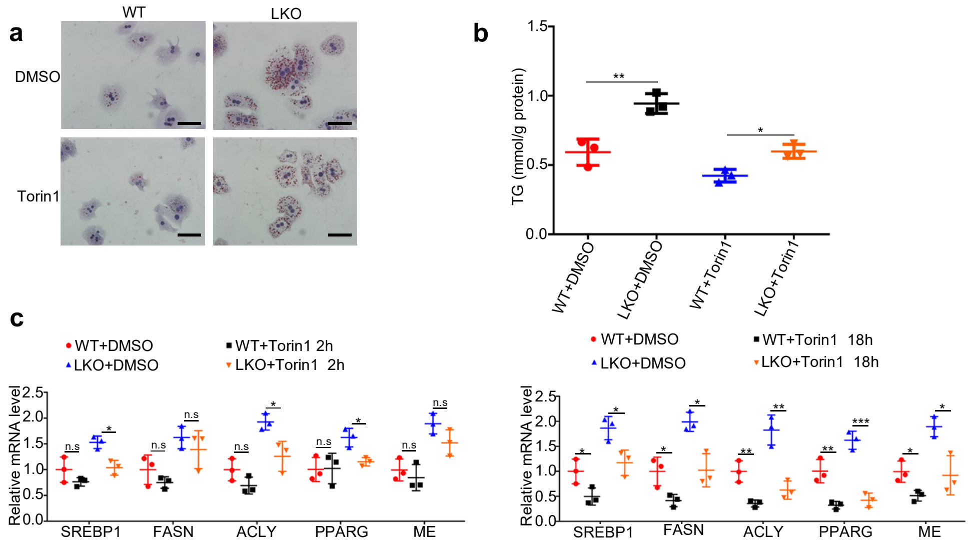

Supplementary Fig. 4 Torin1 treatment reduces lipid deposition in PPDPF-LKO hepatocytes. (a) Oil Red O straining of primary hepatocytes upon Torin1 treatment. Scale bars, 100um.**(b)**Triglyceride (TG) detect of hepatocytes from WT (n=3) and LKO (n=3) mice treated with Torin1 for 24h. Mean±SEM, **p=0.0068 *p=0.011 by two tailed unpaired Student's t-test. **(c)**The mRNA levels of lipid synthesis-associated genes are examined after 2 hours or 18 hours of Torin1 treatment, these cells from primary hepatocytes of WT (n=3) and LKO (n=3) mice. Mean±SEM. See Supplementary Data 3 for statistics. All experiments were repeated 3 times independently.

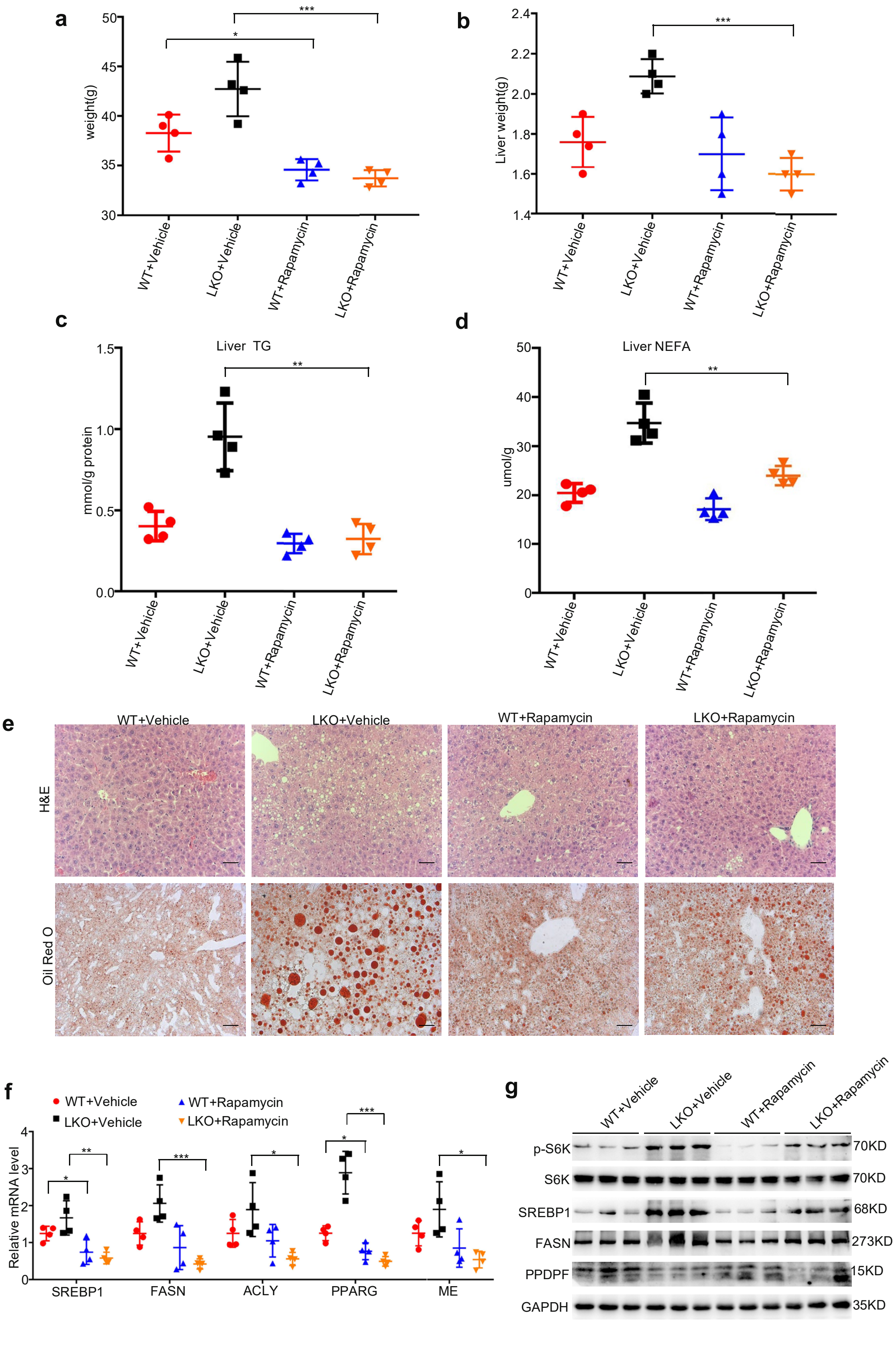

Supplementary Fig.5 Rapamycin treatment inhibits lipid synthesis in PPDPF -LKO mice at 8 months of age on chow diets. (a-d) The body weight(a), liver weight (b), TG (c) and NEFA (d) for each group: WT+Vehicle (n=4), LKO+Vehicle

(n=4), WT+Rapamycin (n=4), LKO+Rapamycin (n=4) under rapamycin treatment at 8 months on chow diets. Mean±SEM, (a) *p=0.0138 ***p=0.0008, (b) ***p=0.0002 , (c) **p=0.0015, (d) **p=0.0032 by two tailed unpaired Student's t-test. **(e)** Representative images of H&E and Oil Red O staining of liver sections from the mice treated with rapamycin at 8 months. Scale bars, 100 um. **(f)** The mRNA expression level of lipogenesis-related genes for each group: WT+Vehicle (n=4), LKO+Vehicle (n=4), WT+Rapamycin (n=4), LKO+Rapamycin (n=4) at 8 months on chow diets. Mean±SEM, *SREBP1*: *p=0.0397 **p=0.0045, *FASN*: ***p=0.0008, *ACLY*: *p=0.0019, *PPARG*: *p=0.0172 ***p=0.0002, *ME*: *p=0.0131 by two tailed unpaired Student's t-test. **(g)** Expression of p-S6K, S6K, SREBP1 and FASN in liver samples after rapamycin treatment. All experiments were repeated 3 times independently.

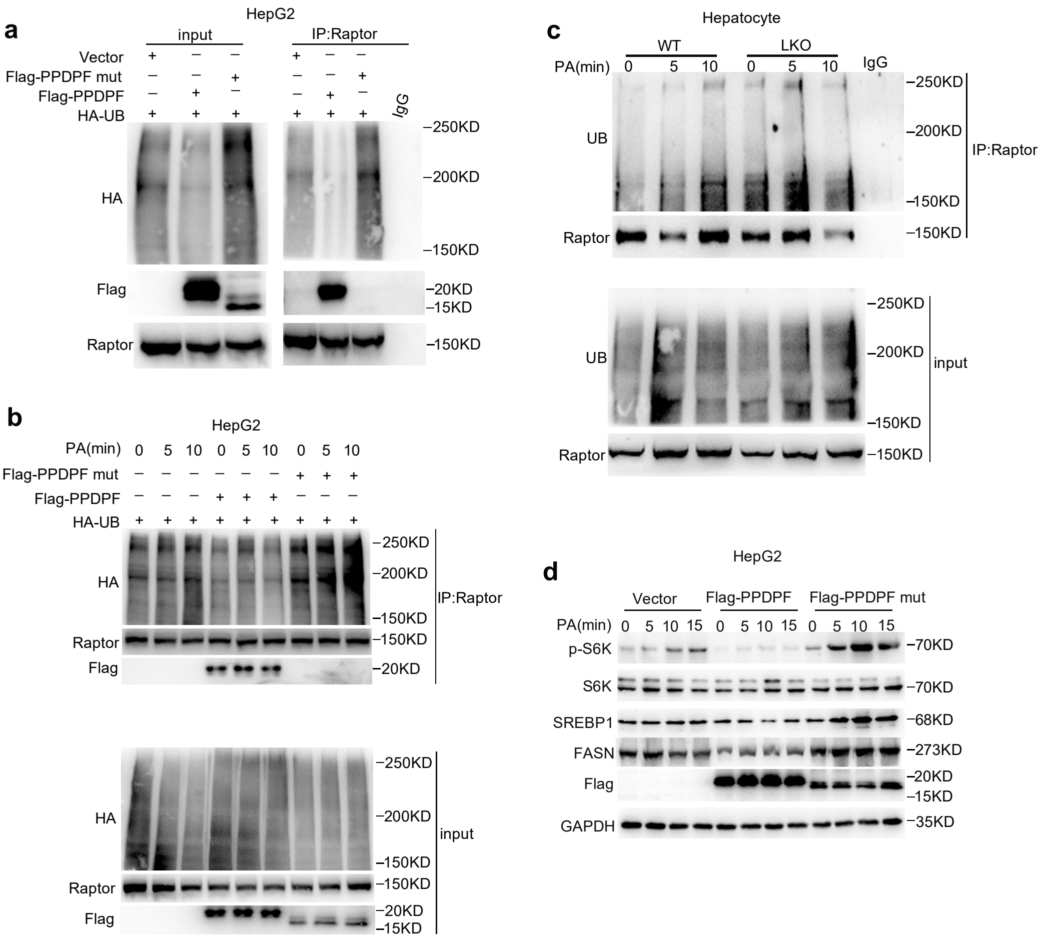

Supplementary Fig.6 Influence of PPDPF and PPDPF mut on Raptor ubiquitination and mTOR signaling pathway. (a) The ubiquitination of Raptor in HepG2 cells was examined. **(b)** The dynamic change of Raptor ubiquitination in HepG2 cells upon PA treatment. **(c)** The ubiquitination of Raptor in WT and LKO hepatocytes was examined. **(d)** Expression of p-S6K, S6K, SREBP1 and FASN in HepG2 cells upon PA stimulation. All experiments were repeated 3 times independently.

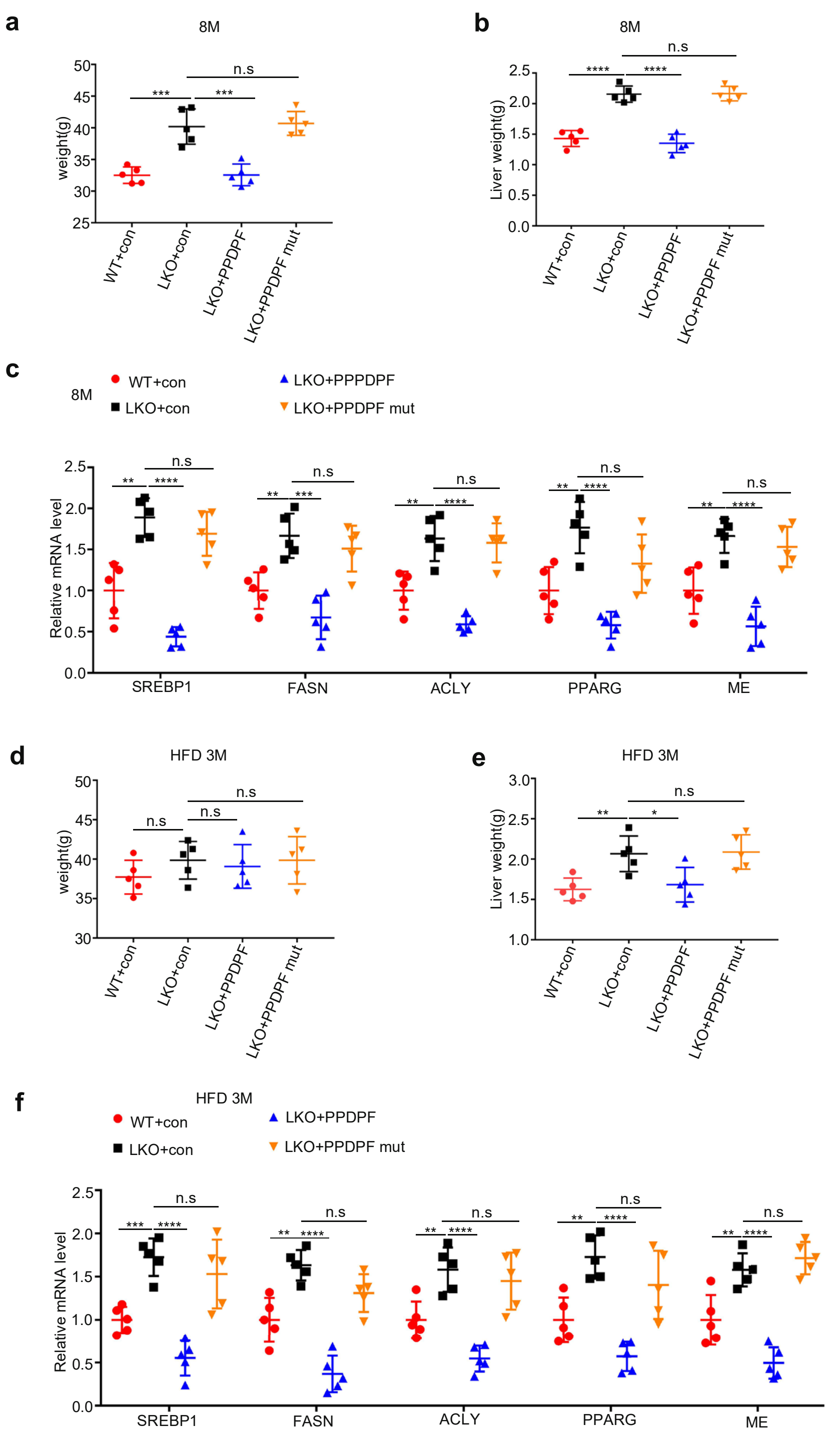

Supplementary Fig.7 AAV8-mediated reintroduction of PPDPF and PPDPF-mut in PPDPF-LKO mice. (a, b) The body weight(a) and liver weight(b) of WT+con, LKO+con, LKO+PPDPF and LKO+PPDPF mut mice(n=5 per group) at 8 months on chow diets. Mean±SEM, n.s (not significant), (a) ***p=0.0005 ***p=0.0008, (b)

****p<0.0001 by two tailed unpaired Student's t-test. **(c)** The mRNA expression level of lipogenesis-related genes of WT+con, LKO+con, LKO+PPDPF and LKO+PPDPF mut mice (n=5 per group) at 8 months on chow diets. Mean±SEM, n.s (not significant), see Supplementary Data 4 for statistics. **(d, e)** The body weight (d), liver weight (e) of WT+con, LKO+con, LKO+PPDPF and LKO+PPDPF mut mice (n=5 per group) at 3 months after HFD feeding. Mean±SEM, n.s (not significant), **p=0.0054 *p=0.0241 by two tailed unpaired Student's t-test. **(f)** The mRNA expression level of lipogenesis-related genes of WT+con, LKO+con, LKO+PPDPF and LKO+PPDPF mut mice (n=5 per group) at 3 months after HFD feeding. Mean±SEM, n.s (not significant), see Supplementary Data 4 for statistics. All experiments were repeated 3 times independently.

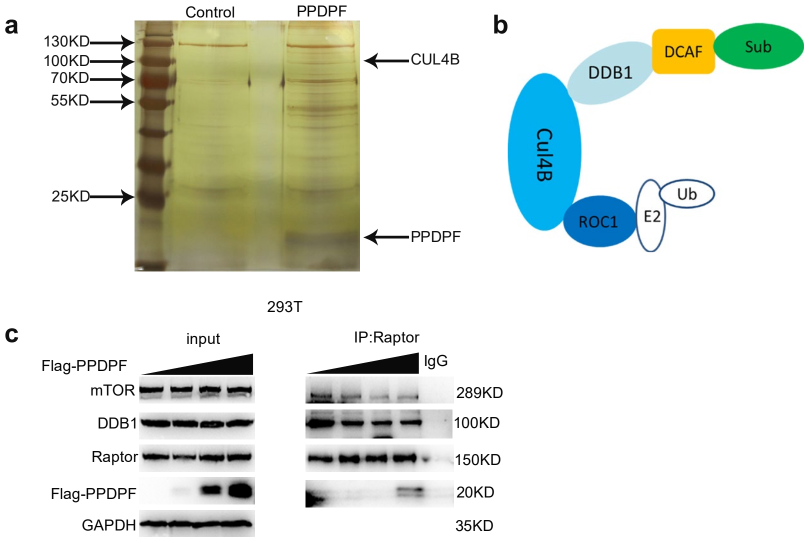

Supplementary Fig.8 The identification of E3 ligase of Raptor. (a) Silver straining of immunoprecipitates of 3xFlag-PPDPF in 293T cells. **(b)** DDB1-CUL4B E3 ubiquitin-ligase complex. **(c)** PPDPF influences Rapor-DDB1 and Raptor-mTOR interaction in a dose-dependent manner. All experiments were repeated 3 times independently.

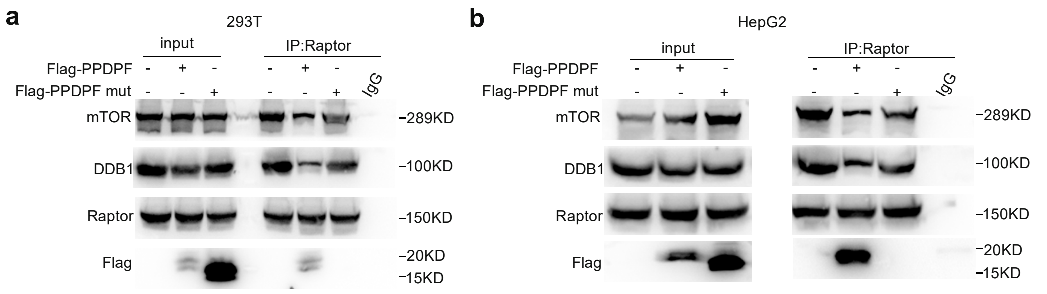

Supplementary Fig.9 PPDPF mutant can not influence DDB1-Raptor interaction. (a,b) Co-immunoprecipitation assay to examine the interaction between PPDPF/PPDPF mut and Raptor in 293T cells **(a)** and HepG2 cells **(b)**. All experiments were repeated 3 times independently.

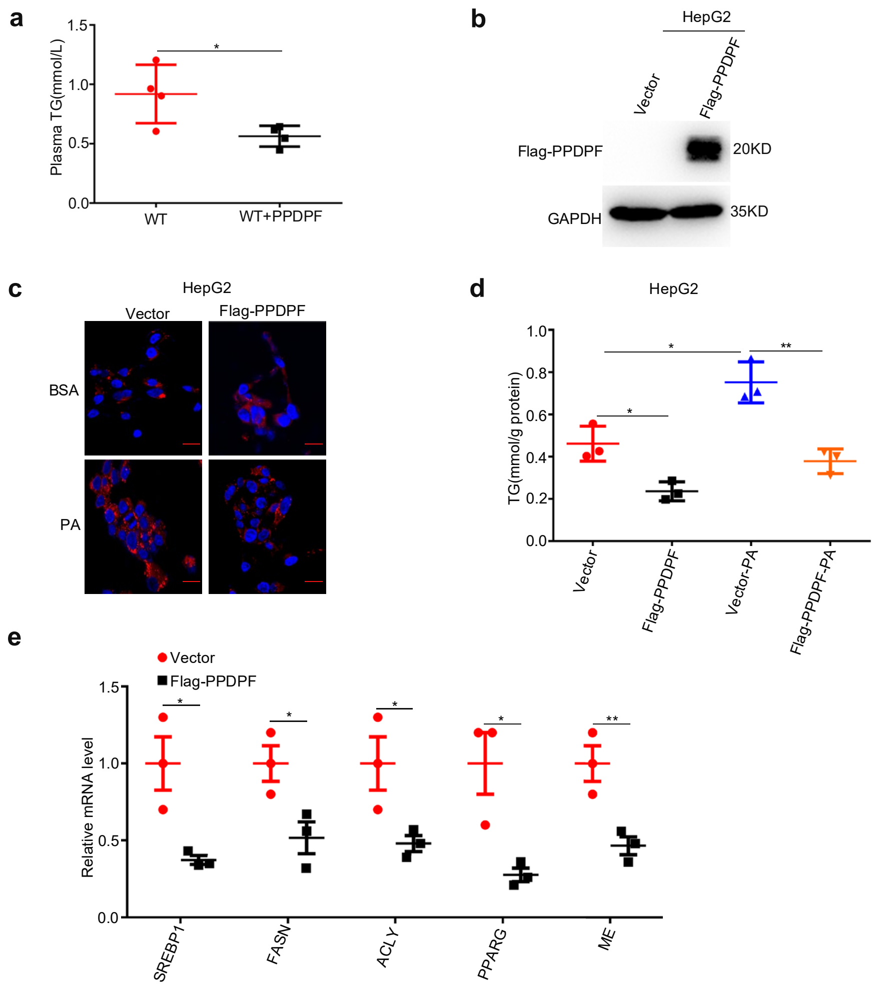

Supplementary Fig.10 Overexpression of PPDPF reduces the lipid deposition in HepG2 cells. (a) Plasma triglyceride (TG) detection of WT (n=4) and WT+PPDPF

(n=4) mice on HFD diet for 3 months. Mean±SEM, *p=0.0346 by two tailed unpaired Student's t-test. **(b)** Western blotting examining the overexpression of PPDPF in HepG2 cells. **(c)** Nile Red staining of HepG2 cells under PA treatment. Scale bars, 100um. **(d)** Triglyceride tests to examine lipid accumulation in HepG2 cells under PA treatment of the control group (n=3) and overexpression group (n=3). Mean±SEM, *p=0.0346 (Vector Vs Flag-PPDPF), **p=0.0047 (Vector Vs Flag-PPDPF-PA), *p=0.0168 (Vector Vs Vector-PA) by two tailed unpaired Student's t-test. **(e)** The relative mRNA levels of the indicated molecules in HepG2 cells of the control group (n=3) and overexpression group (n=3). Mean±SEM, *SREBP1*: *p=0.0234, *FASN*: *p=0.0356, *ACLY*: *p=0.0452, *PPARG*: *p=0.0242, *ME*: **p=0.005 by two tailed unpaired Student's t-test. All experiments were repeated 3 times independently.

Supplementary Table 1. Primary antibodies for Western blotting assay.

Antibody	Cat No.	Manufacturer	Species	Dilution
FASN	3180	CST	Rabbit	1:1000
SREBP1	sc-365514	Santa Cruz	Mouse	1:1000
p-p70 S6K	9234	CST	Rabbit	1:1000
S6K	ab32359	Abcam	Rabbit	1:1000
GAPDH	60004	Proteintech	Mouse	1:3000
Raptor	2280	CST	Rabbit	1:1000
DDB1	11380	Proteintech	Rabbit	1:1000
Flag	F3165	Sigma	Mouse	1:5000
HA	3724	CST	Rabbit	1:1000
Myc	sc-40	Santa Cruz	Mouse	1:1000
Ubiquitin	sc-8017	Santa Cruz	Mouse	1:1000
PPDPF	19912	Proteintech	Rabbit	1:500
GST	6g9	Proteintech	Rabbit	1:1000

Supplementary Table 2. Primers for Real-Time PCR detection.

Gene	Sequence 5’→3’
Mouse SREBF1	F： TGACCCGGCTATTCCGTGA<br>R： CTGGGCTGAGCAATACAGTTC
Mouse FASN	F：GGAGGTGGTGATAGCCGGTAT<br>R：TGGGTAATCCATAGAGCCCAG
Mouse PPARG	F：GGAAGACCACTCGCATTCCTT<br>R：GTAATCAGCAACCATTGGGTCA
Mouse ME	F：GCCGGCTCTATCCTCCTTTG<br>R：TTTGTATGCATCTTGCACAATCTTT
Mouse ACLY	F：ACCCTTTCACTGGGGATCACA<br>R：GACAGGGATCAGGATTTCCTTG
Mouse PPARA	F：AACATCGAGTGTCGAATATGTGG<br>R：CCGAATAGTTCGCCGAAAGAA
Mouse EHHADH	F：ATGGCTGAGTATCTGAGGCTG<br>R：GGTCCAAACTAGCTTTCTGGAG
Mouse MCAD	F：CAAGTTTGCCAGAGAGGAGATTATC<br>R：AACGGGTACTCCCCGCTTT
Mouse ACOX1	F：TAACTTCCTCACTCGAAGCCA<br>R：AGTTCCATGACCCATCTCTGTC

Mouse ACAD1	F：TCTTTTCCTCGGAGCATGACA<br>R：GACCTCTCTACTCACTTCTCCAG
Mouse ECH1	F：AAGATAAGGACGCCATGCTGAA<br>R：TCCAGGTGGCCATGTAGTCA
Mouse CD36	F：ATGGGCTGTGATCGGAACTG<br>R：GTCTTCCCAATAAGCATGTCTCC
Mouse FABP1	F：ATGAACTTCTCCGGCAAGTACC<br>R：GGTCCTCGGGCAGACCTAT
Mouse APOB	F：CGTGGGCTCCAGCATTCTA<br>R：TCACCAGTCATTTCTGCCTTTG
Mouse APOE	F：GCTGGGTGCAGACGCTTT<br>R：TGCCGTCAGTTCTTGTGTGACT
Mouse APOA1	F：GGCACGTATGGCAGCAAGAT<br>R：CCAAGGAGGAGGATTCAAACTG
Human SREBF1	F：ACAGTGACTTCCCTGGCCTAT<br>R：GCATGGACGGGTACATCTTCAA
Human FASN	F：AAGGACCTGTCTAGGTTTGATGC<br>R：TGGCTTCATAGGTGACTTCCA
Human ACLY	F：TCGGCCAAGGCAATTTCAGAG<br>R：CGAGCATACTTGAACCGATTCT
Human PPARG	F：GGGATCAGCTCCGTGGATCT<br>R：TGCACTTTGGTACTCTTGAAGTT
Mouse PPDPF	F：CCACATTCTGCTCTCGTCTC<br>R：AGGCGTCGCCGATAGT
Mouse 18sRNA	F：GTAACCCGTTGAACCCCATT<br>R：CCATCCAATCGGTAGTAGCG
Human 18sRNA	F：GAGAAACGGCTACCACATCC<br>R：CACCAGACTTGCCCTCCA

Supplementary Table 3. Primers for mouse generation.

Gene	Sequence 5’→3’
PPDPF	F： GGCCTTACTCTTGTACTGCTGTC<br>R： GAGAGGATCATGAGCCAGCTTCG

Supplementary Table 4. Primers for plasmid construction.

Plasmids	Sequence Reference	Species	Sequence 5’→3’
P23-3xFlag-PPDPF	NM_024299.4	Human	F：CGGGGTACCATGGCGGCCATCCCCTCCAGCGGCT<br>R：<br>CTAGTCTAGACTGGACGGGGGCCCAGCGCTGGCTGTG
P23-3xFlag-Raptor	NM_020761.3	Human	F：CGGGGTACCATGGAGTCCGAAATGCTGCAATC<br>R：TGCTCTAGAGGTCTGACACGCTTCTCCACCGAGTAC
P23-3xFlag-DDB1	NM_001923.5	Human	F：CGGGGTACCATGTCGTACAACTACGTGGTAACG<br>R：TGCTCTAGAGCATGGATCCGAGTTAGCTCCTCCACA
pcDNA3-HA-PPDPF	NM_024299.4	Human	F：CCGGAATTCATGGCGGCCATCCCCTCCAGC<br>R：CGCGGATCCGGACGGGGGCCCAGCGCTGGCTGTG
pcDNA3.1-MYC-PPDPF	NM_024299.4	Human	F：CGCGGATCCATGGCGGCCATCCCCTCCAGC<br>R：CCGGAATTCGGACGGGGGCCCAGCGCTGGCTGTG
