# Supplementary material for: PPDPF alleviates hepatic steatosis through inhibition of mTOR signaling
Source: Nat Commun. 2021 May 24;12:3059. doi: 10.1038/s41467-021-23285-8 (PMC8144412; doi:10.1038/s41467-021-23285-8)
Supplement: Supplementary file 3 — Description of Additional Supplementary Information [file 41467_2021_23285_MOESM3_ESM.pdf]

Description of Additional Supplementary Files

File Name: Supplementary Data 1

Description: The statistical analysis for Fig.2e.

File Name: Supplementary Data 2

Description: The statistical analysis for Fig.8f and Fig.8g.

File Name: Supplementary Data 3

Description: The statistical analysis for Supplementary Fig.4c.

File Name: Supplementary Data 4

Description: The statistical analysis for Supplementary Fig.7c and Supplementary Fig.7f
